# Mitogen and Stress Activated Kinases Act Co-operatively with CREB during the Induction of Human Cytomegalovirus Immediate-Early Gene Expression from Latency

**DOI:** 10.1371/journal.ppat.1004195

**Published:** 2014-06-12

**Authors:** Verity G. Kew, Jinxiang Yuan, Jeffery Meier, Matthew B. Reeves

**Affiliations:** 1 Department of Medicine, Addenbrooke's Hospital, University of Cambridge, Cambridge, United Kingdom; 2 Department of Medicine, University of Iowa, Iowa City, Iowa, United States of America; 3 Institute of Immunity & Transplantation, Division of Infection & Immunity, Royal Free Hospital, University College London, London, United Kingdom; Cleveland Clinic Foundation, United States of America

## Abstract

The devastating clinical consequences associated with human cytomegalovirus (HCMV) infection and reactivation underscores the importance of understanding triggers of HCMV reactivation in dendritic cells (DC). Here we show that ERK-mediated reactivation is dependent on the mitogen and stress activated kinase (MSK) family. Furthermore, this MSK mediated response is dependent on CREB binding to the viral major immediate early promoter (MIEP). Specifically, CREB binding to the MIEP provides the target for MSK recruitment. Importantly, MSK mediated phosphorylation of histone H3 is required to promote histone de-methylation and the subsequent exit of HCMV from latency. Taken together, these data suggest that CREB binding to the MIEP is necessary for the recruitment of the kinase activity of MSKs to initiate the chromatin remodelling at the MIEP required for reactivation. Thus the importance of CREB during HCMV reactivation is to promote chromatin modifications conducive for viral gene expression as well as acting as a classical transcription factor. Clearly, specific inhibition of this interaction between CREB and MSKs could provide a strategy for therapeutic intervention.

## Author Summary

Human cytomegalovirus (HCMV) infection of immune-compromised individuals is a significant cause of morbidity. In a number of settings disease is caused by the reactivation of a pre-existing quiescent infection of the host (latency) which, in the absence of a controlling immune response, is a major source of disease. Work to understand the switch from a latent to active (reactivated) infection has focussed on the regulation of the promoter that controls the major viral gene products IE72 and IE86 – an important first step towards HCMV reactivation. In this study we have correlated the activation of cellular signalling pathways with the downstream activation of this promoter. Specifically, the activation of cellular kinase in an ERK-MAPK dependent manner which displays an affinity for a protein bound to a key viral promoter drives a change in the chromatin architecture that allows viral gene expression – releasing the virus from the latent state.

## Introduction

A defining characteristic of the herpesvirinae is the establishment of lifelong latent infection of the host following a primary challenge. The prototype betaherpesvirus member, Human Cytomegalovirus (HCMV) is no exception, establishing a latent reservoir in the haematopoietic cells of the bone marrow (reviewed in [1]). It is likely that sporadic reactivation and shedding – although subclinical – is important for the sero-prevalence of HCMV in the population via horizontal transmission. However, it is the reactivation of HCMV (as well as primary infection) in immune-compromised transplant recipients or late-stage AIDS sufferers that represent a significant source of morbidity and mortality in these high risk patient populations [2], [3], [4]. Consequently, an understanding of the molecular and physiological cues that promote reactivation could inform on the design of novel therapeutic strategies.

Studies from a number of laboratories using analyses of experimental and natural latency have led to a consensus that HCMV establishes latency in the haematopoietic cells of the bone marrow [5], [6], [7], [8], [9], persists in the monocyte/myeloid lineage [10], [11], [12] and reactivates upon the differentiation and/or stimulation of these cells to a more mature or activated phenotype [8], [13], [14], [15], [16], [17], [18]. Furthermore, these reactivation events appear to be augmented by inflammatory cytokine signalling (e.g. TNF, interferon-gamma) acting in concert with cellular differentiation [13], [16], [18]. Indeed we, and others, have reported that the incubation of latently infected myeloid cells with IL-6 can be a trigger for HCMV reactivation in experimental latency [13], [17]. Furthermore, we have also observed that the reactivation of naturally latent virus from the dendritic cells (DCs) of healthy individuals can be markedly abrogated using neutralising IL-6 antibodies [13], [16]. Although we have postulated a role for ERK-MAPK and interleukin-6 signalling in this event the underlying mechanisms are still unclear [13].

In concert with observations in primary cells, studies of the quiescent infection of the embryonal T2 carcinoma cell line have suggested that elevated cAMP signalling promotes reactivation in a creb response element (CRE) dependent manner [19], [20]. This alleviation of quiescent infection can be achieved using both chemical (forskolin) and biological (vasoactive intestinal peptide) activity and is suggestive that regions of the major immediate early promoter (MIEP) shown to be redundant during lytic infection may have important functions during reactivation from latency in response to these stimuli [19], [20], [21]. In contrast, transfection experiments with the MIEP point towards a role for TNF-α and downstream NF-ĸB signalling during reactivation [22] and, indeed, murine CMV (MCMV) studies support an important role for these signalling pathways also [23], [24], [25]. Furthermore, the reactivation of HCMV from experimentally latent cell lines has also been reported using phorbol esters and TNF-α - again consistent with a role for aspects of NF-ĸb signalling being involved in HCMV reactivation [26], [27] by triggering IE gene expression.

ERK MAPK signalling can be activated by a diverse number of ligands acting through two upstream modulators. Classical Ras-Raf mediated ERK-MAPK signalling often acts in response to mitogenic stimulation [28] with aberrant signalling associated with a transformed phenotype in oncogenic cells [29]. Alternatively, signalling can be activated by tpl2/Cot1 [30] which targets downstream ERK phosphorylation following the engagement of Toll-like [31] and receptors in the TNF super-family [32]. ERK phosphorylation promotes nuclear translocation and subsequent phosphorylation of a number of target proteins [33]. These targets include the mitogen and stress activated protein kinases (MSKs) and 90kDa ribosomal S6 kinase (RSKs) [34], [35], [36] which themselves target a number of cellular substrates [35], [37]. Interestingly, these targets include transcription factors and histone proteins that may have roles in the regulation of HCMV immediate early gene expression [15], [19], [20], [38], [39], [40].

In this study, we have sought to address the mechanisms that are important for the induction of IE gene expression required to initiate HCMV reactivation. We have focussed on the interplay between cellular signalling pathways and the activation of target transcription factors. Building on our previous studies looking at ERK-MAPK signalling we have addressed the impact of CREB binding to the MIEP and how it asks as a platform to integrate cellular signalling at the MIEP in differentiating DCs capable of supporting reactivation. Using interleukin-6 or LPS as a tool to initiate HCMV reactivation, we have identified that phosphorylated CREB occupancy on the MIEP is important for the induction of IE gene expression. Furthermore, we also show that histone H3 phosphorylation at serine 10 correlates with induction of IE gene expression. Interestingly, the activation of the upstream MSKs is pivotal for reactivation - with CREB and Histone H3 being major targets for this kinase which is also recruited to the MIEP prior to the induction of IE gene expression. Intriguingly, we also observe that histone H3 phosphorylation at the MIEP is defective in the ΔCRE virus suggesting that binding and phosphorylation of CREB by MSKs promotes the subsequent phosphorylation of histone H3. Finally, we show that abrogation of ERK-MAPK signalling using a dominant negative form of MEK1, the upstream kinase of ERK1/2, diminishes MSK activation and blocks CREB and histone H3 phosphorylation at the MIEP resulting in a substantial defect in IE gene expression. Since histone H3 phosphorylation is a key event in the transition from a repressed (latent) to an active (reactivated) promoter these data suggest that CREB is an important factor for reactivation not only because of its classical role as a transcriptional activator but also via co-operative interaction with MSK to drive elevated histone phosphorylation at the MIEP and subsequent activation of gene expression.

## Results

### IE gene expression from latent virus is promoted through Ras-Raf mediated ERK-MAPK signalling

**Figure 1:**
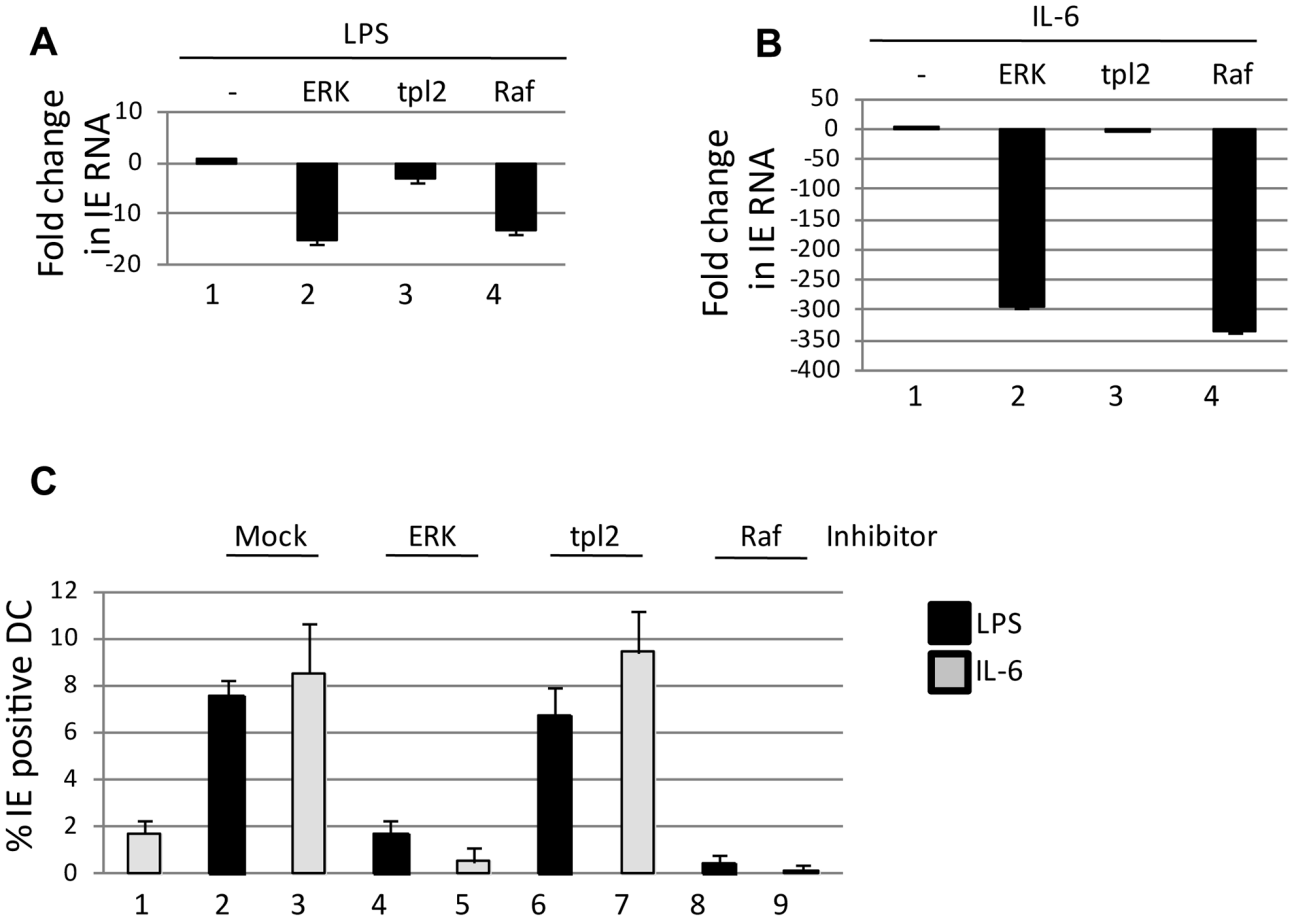
Signalling via the Ras-RAF arm of ERK-MAPK is required for HCMV reactivation. **A–B**) RNA isolated from immature DCs or DCs stimulated with LPS (A) or IL-6 (B) was amplified qRT-PCR (IE and GAPDH). Prior to addition of LPS or IL-6 cells were incubated with inhibitors of ERK, Raf or tpl2 signalling for 1 hour. IE RNA expression was expressed as a fold decrease compared to mock treated control **C**) immature DCs (1) were incubated with DMSO (2,3) or ERK (4,5), tpl2 (6,7) and Raf (8,9) inhibitors for 1 hour then stimulated with LPS (black) or IL-6 (grey) to promote reactivation. The percentage of IE positive cells was calculated by indirect immunofluorescence and Hoechst nuclear counterstaining. S.D. shown from n = 3 (A–C).

Ongoing studies of the pathways important for HCMV reactivation from DCs have highlighted the ERK-MAPK pathway as an important factor [13]. The maturation of DCs in response to LPS triggers ERK phosphorylation which could occur via at least two potential pathways – tpl2/Cot1 or Ras-Raf. To test which pathway was important in the context of LPS induced reactivation, latently infected monocytes were differentiated to immature DCs and then, prior to stimulation with LPS, incubated with a pan ERK-MAPK inhibitor or specific inhibitors of the tpl2 and Ras-Raf pathways. A qRT-PCR analysis for IE gene expression confirmed our previous data that ERK-MAPK inhibition blocked induction of HCMV IE gene expression (Fig. 1A,B). However, inhibition with the upstream targets suggested that it was Ras-Raf, and not tpl2 signalling, which was important for triggering the induction of IE RNA expression (Fig. 1A,B). Previously, we have observed that a major component of HCMV reactivation upon DC stimulation with LPS is IL-6 and that activation of ERK-MAPK signalling was also important in this response. Again, consistent with a major role for Ras-Raf it was only inhibition of this effector molecule – and not tpl2 signalling – which had a major impact on HCMV IE RNA reactivation in response to IL-6 (Fig. 1B). Crucially, this defect in RNA expression was also observed at the protein level. Pre-incubation of immature DCs with inhibitors prior to stimulation with LPS or IL-6 resulted in a marked defect in the number of IE-expressing cells in ERK, Ras-Raf but not tpl2 inhibitor treated cells (Fig. 1C). Importantly, the observation that LPS induced gene expression is dependent on Ras signalling is consistent with LPS inducing reactivation via an indirect mechanism (IL-6; Ras-ERK signalling) rather than a direct mechanism (tpl2-ERK signalling).

### CREB binding and phosphorylation to the MIEP is important for the induction of IE gene expression in primary DCs

**Figure 2:**
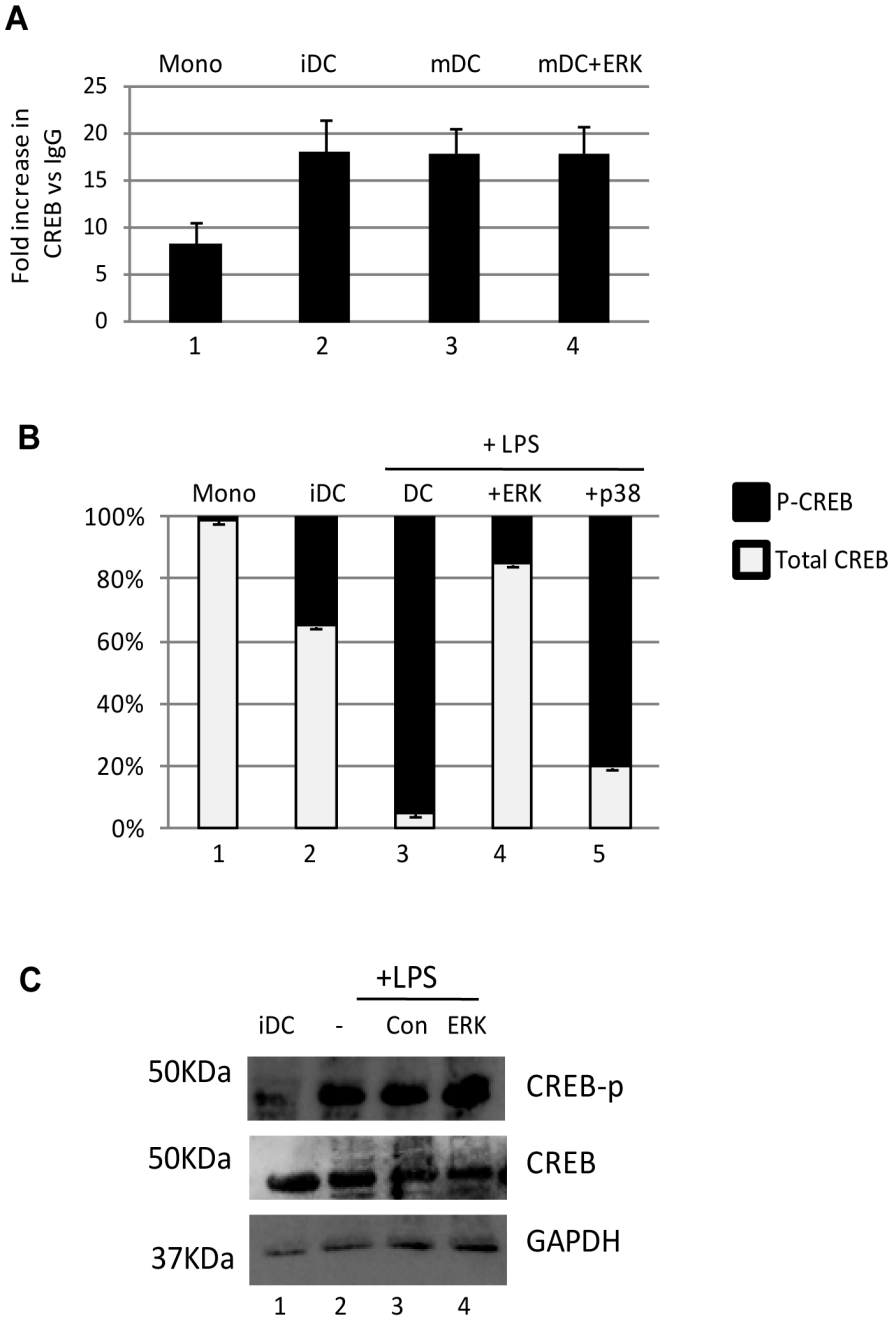
The binding of phosphorylated CREB to the MIEP correlates with reactivation. **A**) Chromatin immunopreciptations on monocytes (Mono), immature DCs (iDC), mature DCs (mDC) or mature DCs pre-treated with ERK inhibitor were performed with an anti-CREB or isotype control antibody. CREB binding is expressed relative to IgG control. **B**) Chromatin immunopreciptations on monocytes (Mono), immature DCs (iDC), mature DCs (mDC), mature DCs pre-treated with ERK or p38 inhibitors were performed with an anti-CREB, anti-phospho CREB or isotype control antibody. The relative levels of phosphorylated CREB binding was expressed a percentage of total CREB binding. S.D. shown from n = 3 (A,B). **C**) Western blot for phosphor-CREB, total CREB and GAPDH was performed on immature DCs or DCs stimulated with LPS after incubation with mock, DMSO or ERK-MAPK inhibitor.

Studies with the laboratory strain, Towne, using a quiescent infection model in T2 cells have suggested that the CREB response elements in the MIEP could have an important role in reactivation in response to mitogenic stimuli [20] and thus we hypothesised that differential binding to the MIEP of CREB could be a potential mechanism in primary DCs. To address this, a series of experiments were performed in reactivating CD14+ derived DCs. (Fig. 2). Firstly we examined CREB binding to the MIEP. Interestingly, the co-immunoprecipitation of the MIEP with CREB was observed in both CD14+ monocytes as well as differentiated DCs although increased levels of specific CREB binding was detected as the cells became more differentiated when compared to the isotype matched control (Fig. 2A). However, despite the inhibitory effect (Fig. 1), pre-treatment with an ERK-MAPK had no impact on the binding of CREB to the MIEP (Fig. 2A) suggesting that CREB binding to the MIEP was not inhibited by ERK. However, a second analysis that assayed relative levels of CREB and phosphorylated CREB (p-CREB) bound to the MIEP suggested that the levels of CREB phosphorylation most strongly correlated with induced IE gene expression – and the effects of chemical inhibitors. In CD14+ monocytes, minimal binding of p-CREB to the MIEP was observed. In contrast, in reactivating cells the predominant form was p-CREB except when the cells were pre-incubated with an ERK-MAPK inhibitor (Fig. 2B) at a concentration known to block induction of HCMV IE gene expression (Fig. 1). Furthermore, Western blot analysis suggested that these effects were not due to a global inhibition of CREB phosphorylation in the cell by the ERK inhibitor (Fig. 2C) consistent with multiple pathways capable of promoting phosphorylation of the total cellular pool of CREB. Taken together these data suggested that whilst inhibition of ERK-MAPK signalling had little impact on CREB binding to the MIEP it did result a marked reduction in the detection of p-CREB bound to the MIEP correlating with a defect in the levels of viral IE gene expression.

### The CRE elements in the MIEP of HCMV promote efficient IE gene expression upon exit from latency

**Figure 3:**
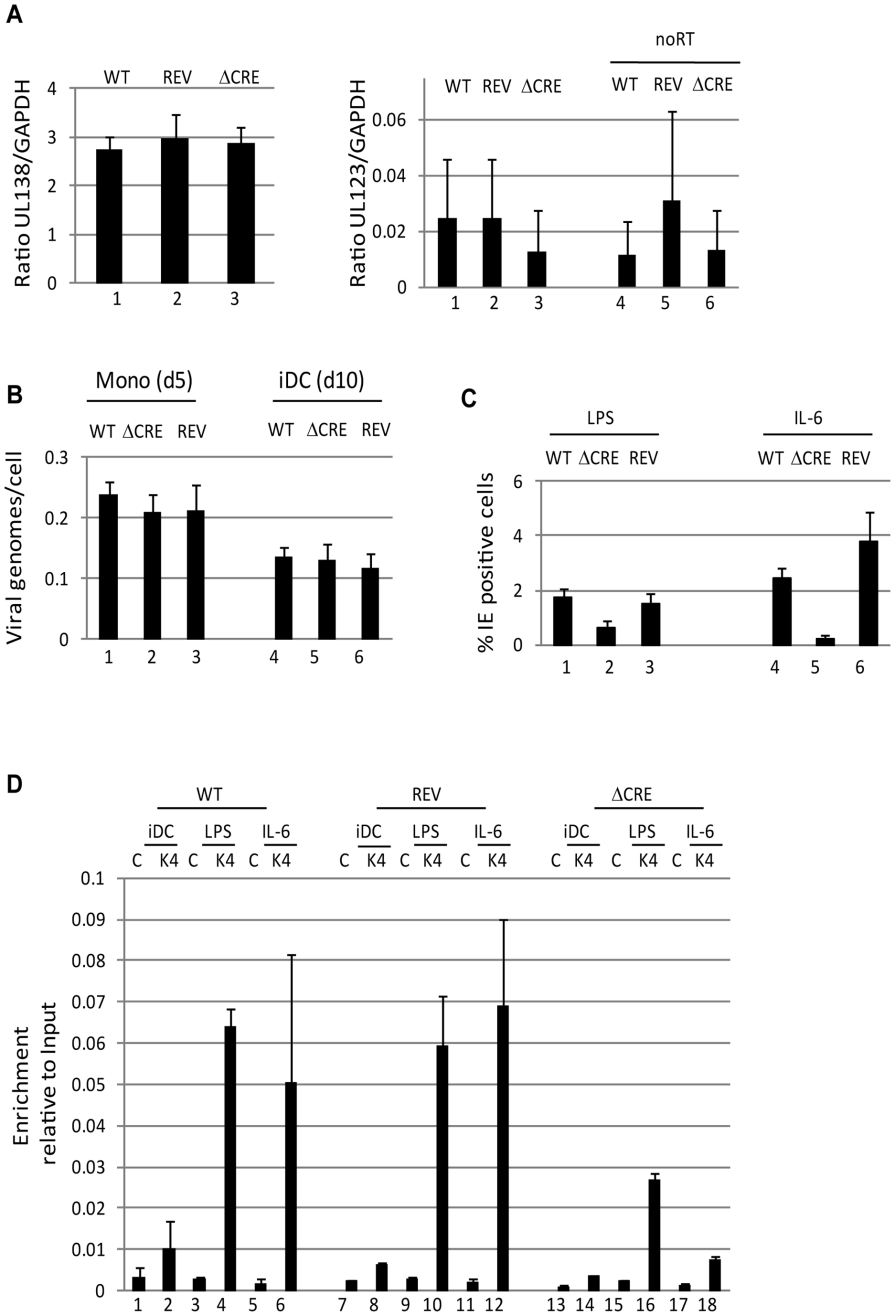
Deletion of the creb response elements from the MIEP abrogates reactivation in DCs. **A**) Monocytes infected with wild type (WT), Revertant (Rev) or CRE deletion virus (ΔCRE) were analysed 5 days post infection for UL138, UL123 and GAPDH gene expression by qRT-PCR. Viral gene expression (UL138 & UL123) was expressed as a ratio to GADPH. A further no RT control is shown for UL123 (4–6) to discriminate between RNA and low level DNA contamination. **B**) DNA from 10∧5 latently infected cells was harvested and viral DNA quantified by qPCR. After normalisation to GAPDH, viral genome copy number per cell was calculated. **C**) immature DCs derived from HCMV infected monocytes were stimulated with LPS (1–3) or IL-6 (4–6) to promote reactivation. The percentage of IE positive cells was calculated by indirect immunofluorescence and Hoechst nuclear counterstaining. S.D. shown from n = 3 (A,B). **D**) Chromatin immunopreciptations on immature DCs (iDC) derived from monocytes infected with wild type (WT), Revertant (Rev) or CRE deletion virus (ΔCRE) were performed alongside LPS (2,5,8) and IL-6 (3,6,9) stimulated DCs (3 hours post stimulation) for histone H3-K4^2Me^ binding. DNA was amplified in an MIEP PCR and expressed as ratio to input. S.D. of n = 2.

The prediction therefore would be that deletion of the CRE within the MIEP would impact on HCMV reactivation from DCs. To test this, 19 bp repeat deletions were generated in the endothelial-tropic strain, TB40/e as described previously for Towne [21]. Firstly, we confirmed that CREB did not bind to the MIEP of the ΔCRE virus (Fig. S1). Then these viruses were used to establish a latent infection in CD14+ monocytes as evidenced by long term UL138 gene expression in an absence of UL123 expression. Unsurprisingly, there appeared to be no overt defect in the ability of the mutant virus to establish a latent infection nor any differential ability to carry HCMV genomes from monocytes to immature DCs (Fig. 3A,B). However, when the cells were differentiated and then subsequently stimulated with LPS or IL-6 a defect in the ability of the ΔCRE mutant virus re-initiate IE gene expression was observed (Fig. 3C) – an effect more profound when IL-6 stimulation was used (Fig. 3C). These data directly correlated with a ChIP analysis for the presence of histone 3 dimethylated at lysine 4 (H3-K4^2me^) at the MIEP which, indicative of active transcription from a promoter, was more abundant on the wild type and revertant MIEPs compared with the ΔCRE (Fig. 3D). The defect in MIEP activity during reactivation appeared to be specific for this phase of infection as the initiation of lytic infections in both HFFs and DCs was indistinguishable between the mutant and wild type (Fig. S2). In contrast to our observations with the ΔCRE virus, a deletion of the NF-ĸB binding sites from the MIEP appeared to have little impact on the induction of IE gene expression in primary DCs in response to IL-6 (Fig. S3). Thus, under these experimental conditions, the CRE sites in the MIEP were important for the induction of IE gene expression upon exit from latency.

### Inhibition of mitogen and stress activated protein kinase activity blocks induction of HCMV gene expression

**Figure 4:**
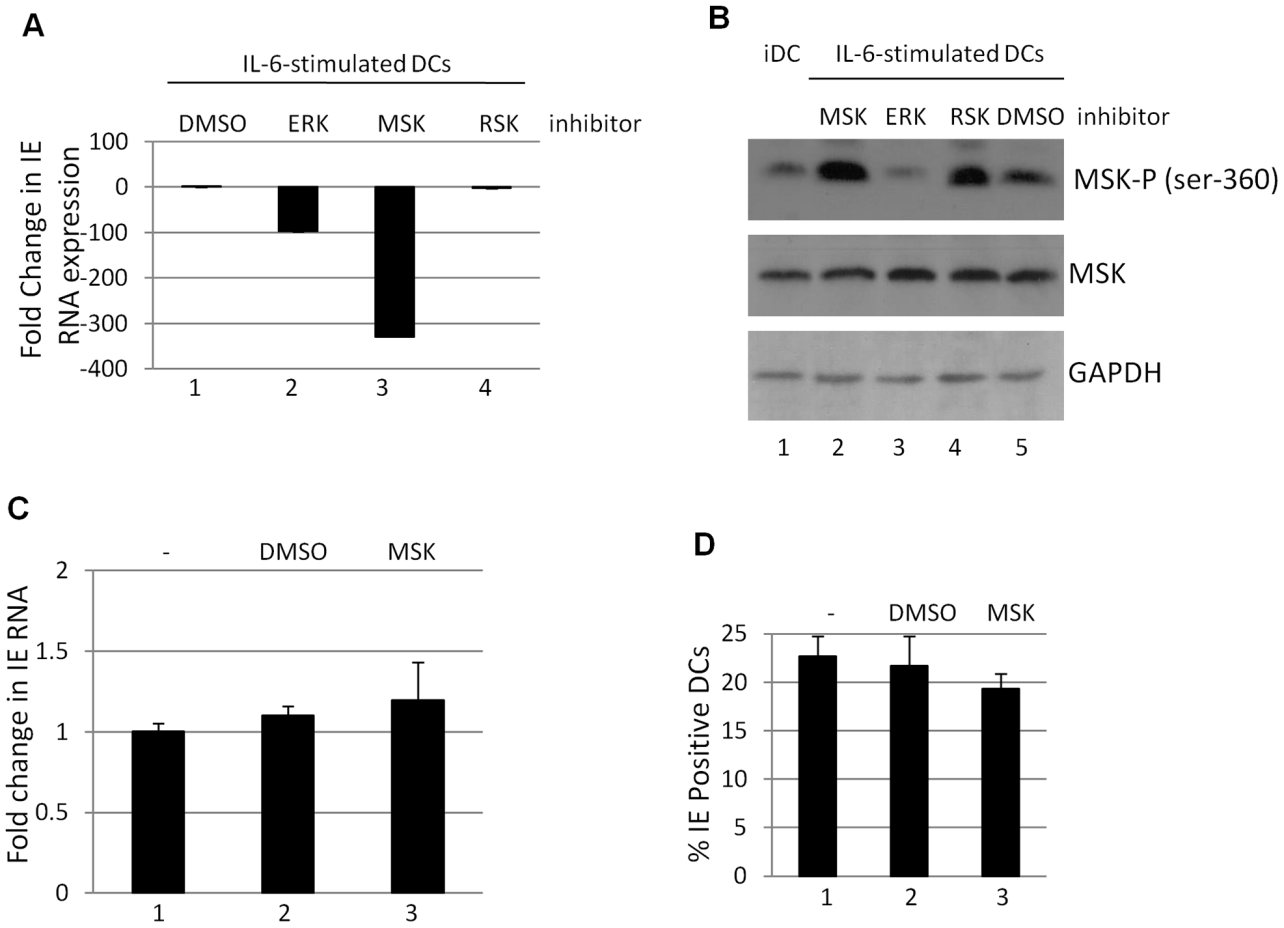
Inhibition of mitogen and stress activated kinase activity is a potent inhibitor of HCMV reactivation. **A**) RNA isolated from DCs stimulated with IL-6 (24 hours) following incubation DMSO (1) or ERK (2), MSK (3) and RSK (4) inhibitors was amplified by qRT-PCR. IE expression was standardised to GAPDH (2^ΔCT^) and the fold change in gene expression (2^ΔΔCT^) was expressed relative to the DMSO sample. **B**) Western blot analysis for phosphor- and total MSK was performed on iDCs or iDCs stimulated with IL6 following pre-treatment with RSK, ERK, MSK inhibitors or DMSO control. **C**) RNA isolated from DCs pre-treated with mock (1), DMSO (2) or MSK inhibitor (3) then infected for 8 hours with HCMV (HFF MOI = 5) was analysed for IE expression by qRT-PCR. **D**) DCs pre-treated with mock (1), DMSO (2) or MSK inhibitors (3) were infected with HCMV (HFF MOI = 5). Twenty four hours post infection the percentage of IE positive cells was calculated by indirect immunofluorescence and Hoechst nuclear counterstaining. S.D. shown from n = 3 (A–C).

Taken together these data suggested that ERK-MAPK signalling initiated by mitogenic stimuli (i.e. IL-6) promoted HCMV reactivation in a Ras-Raf dependent manner and that CREB, a downstream target of ERK, was important for this. Thus our next aim was to identify downstream events associated with ERK-MAPK signalling that could be important for HCMV gene expression and, consequently, our interest focussed on the MSK and RSK families of proteins – major contributors to ERK signal transduction [35], [37]– which target a number of proteins that have been linked with HCMV gene expression including CREB [41]. We first asked whether inhibition of either of these pathways resulted in an inhibition to HCMV reactivation. Pre-incubation of immature DCs with an MSK inhibitor significantly abrogated IL-6 induced IE gene expression in reactivating DCs (Fig. 4A). Interestingly, this effect was more profound than observed with an ERK-MAPK inhibitor (possibly due to the incomplete inhibition of MSK phosphorylation seen with the ERK-MAPK inhibitor (Fig. 4B)). Furthermore, the effect was specific for the MSK family as no effect was observed with an RSK inhibitor (Fig. 4A). Furthermore, the effects appeared to be specific to latency. Primary infection of DCs in the presence or absence of MSK or RSK inhibitors had little impact on IE gene expression when measured by IE-RT-PCR (Fig. 4C) or by indirect immuno-fluorescent staining for IE protein expression (Fig. 4D).

### Mitogen and stress activated protein kinase activation is important for CREB and histone H3 phosphorylation at the MIEP

**Figure 5:**
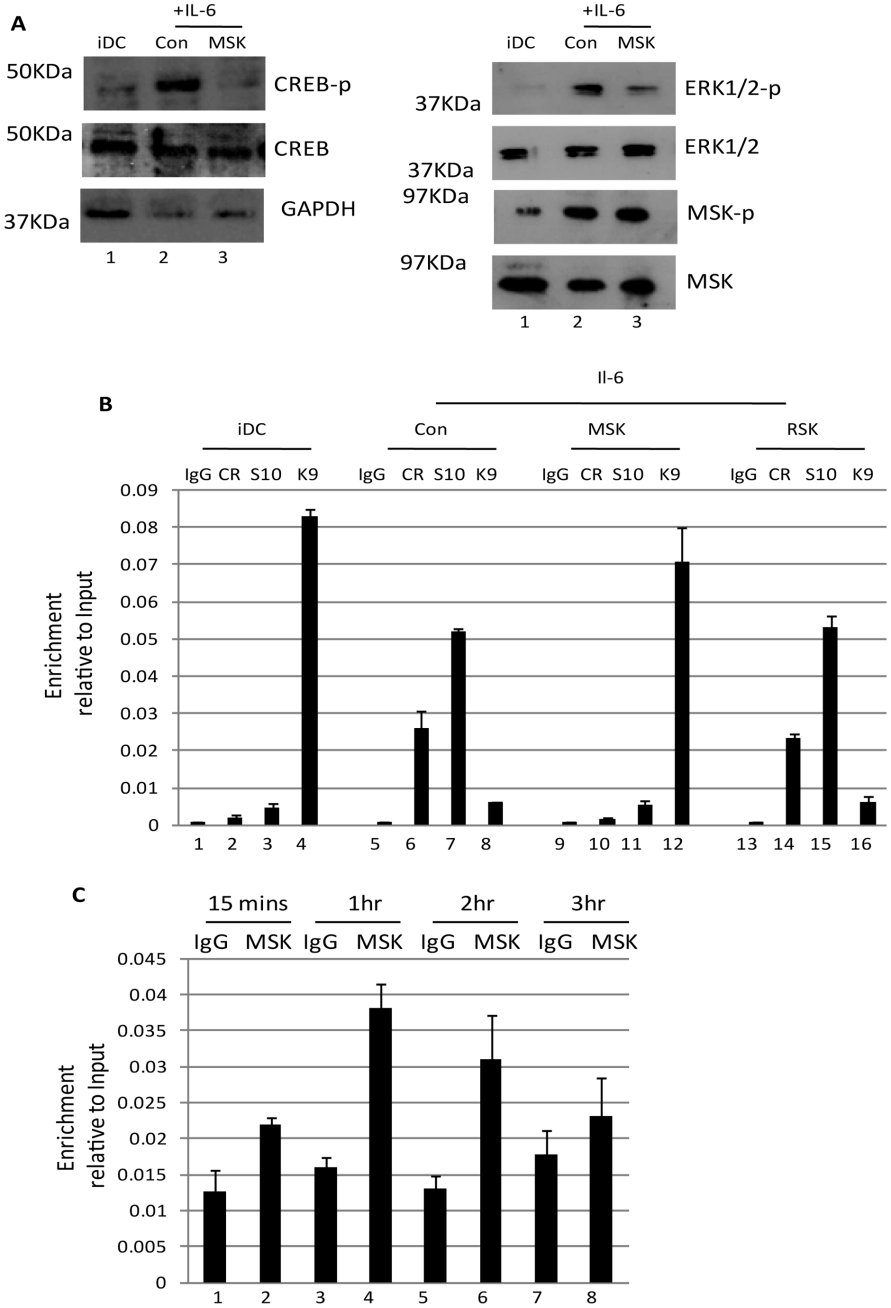
Inhibition of mitogen and stress kinase activity blocks CREB and histone phosphorylation at the MIEP. **A**) Western blot for phosphor and total CREB, phosphor and total ERK1/2, phosphor and total MSK and GAPDH was performed on immature DCs or DCs stimulated with IL-6 (30 mins) after incubation with DMSO or MSK inhibitor (2 hours). **B**) Chromatin immunopreciptations on immature DCs (iDC) derived from monocytes infected with HCMV (1–3) were performed alongside IL-6 (4–12) stimulated DCs for phosphor-CREB (CR), histone H3-S10^P^ (S10) and histone H3-K9^3Me^ (K9) binding at 2 hours post stimulation. DNA was amplified in an MIEP PCR and expressed as ratio of the Input sample. S.D. of n = 2. **C**) Chromatin immunopreciptations on immature DCs (iDC) stimulated with IL-6 were performed at 15 mins to 3 hours post stimulation with an anti-MSK antibody or an isotype matched control. DNA was amplified in an MIEP PCR and expressed as ratio of the Input sample. S.D. of n = 2.

A pervading theme throughout these and other studies is that elements associated with reactivation either increase or decrease the relative efficiency of reactivation. As such, we remained intrigued by the observation that MSK inhibitors had such a dramatic impact on HCMV IE gene expression in DCs (Fig. 4) and thus we reasoned it was an essential integration point for defining the ERK triggered response. Indeed, unlike ERK-MAPK, the inhibition of MSK activity profoundly blocks IL-6 induced CREB phosphorylation in DCs (Fig. 5A) whilst, unsurprisingly, having little impact on the phosphorylation of ERK and MSK themselves (Fig. 5A). Although we cannot rule out an effect of H-89 on Protein Kinase A activity – itself a protein that can phosphorylate CREB – the rapid induction of MSK phosphorylation by IL-6 which signals in a PKA independent manner [42], [43].

Consequently, we hypothesised that other targets of MSK may also be important for inducing IE gene expression from latency. An attractive candidate was histone H3. Predominantly methylated when bound to the MIEP in latency, reactivation in DCs is concomitant with global acetylation of histones bound to the MIEP [15]. Clearly signalling pathways dictate this phenotypic switch and we focussed on the ability of MSK to promote histone H3 phosphorylation at serine residue 10 [44] – an event which has been hypothesised to provide a critical intermediary step between histone de-methylation and subsequent histone acetylation on multiple ‘early response’ cellular promoters [45], [46].

A time of addition study, analysing the ability of neutralising IL-6 antibodies to block HCMV reactivation following stimulation with LPS, revealed that the effects of IL-6 signalling must manifest at immediate-early times post infection (Fig. S4). Consequently, we performed an initial time-course analysis revealed that histone phosphorylation at serine-10 occurred rapidly with phosphorylation detectable within 1 hour post stimulation (Fig. S5). Consistent with a role for ERK-MAPK activation in reactivation we also observed that histone phosphorylation and acetylation was observed in IE-expressing DCs and that this phenotype was inhibited by ERK-MAPK or Raf inhibitors (Fig. S6). Furthermore, the phosphorylation of histone H3 in response to IL-6 did not require de novo viral (or cellular) gene expression (Fig. S7) suggesting it occurred prior to HCMV lytic gene expression and thus reactivation. More pertinently, the pre-incubation of immature DCs with MSK inhibitors resulted in a significant decrease in the detection of the phosphorylated forms of both histone H3 and CREB bound to the MIEP (Fig. 5B). In contrast, use of an RSK inhibitor appeared to have no impact (Fig. 5B). Furthermore, the relatively higher levels of H3-K9 methylation at the MIEP in MSK inhibitor treated cells supports the hypothesis that MSK activity is required for a switch in the chromatin phenotype from a repressed to active state at the MIEP.

Finally, we examined reactivating cells for the presence of MSK at the MIEP. A time-course analysis showed that MSK immune-precipitated with the MIEP but, unlike observed with histone H3 phosphorylation (Fig. S5), the occupancy appeared far more transient (Fig. 5C) which is likely reflected in the different functional roles these proteins play. Both histones and CREB are likely associated with the MIEP for longer duration whereas MSK recruitment and activity is likely a more acute response to signalling. Once the targets are phosphorylated MSK may be released from the complex.

### The phosphorylation of CREB bound to the MIEP promotes histone H3 phosphorylation

**Figure 6:**
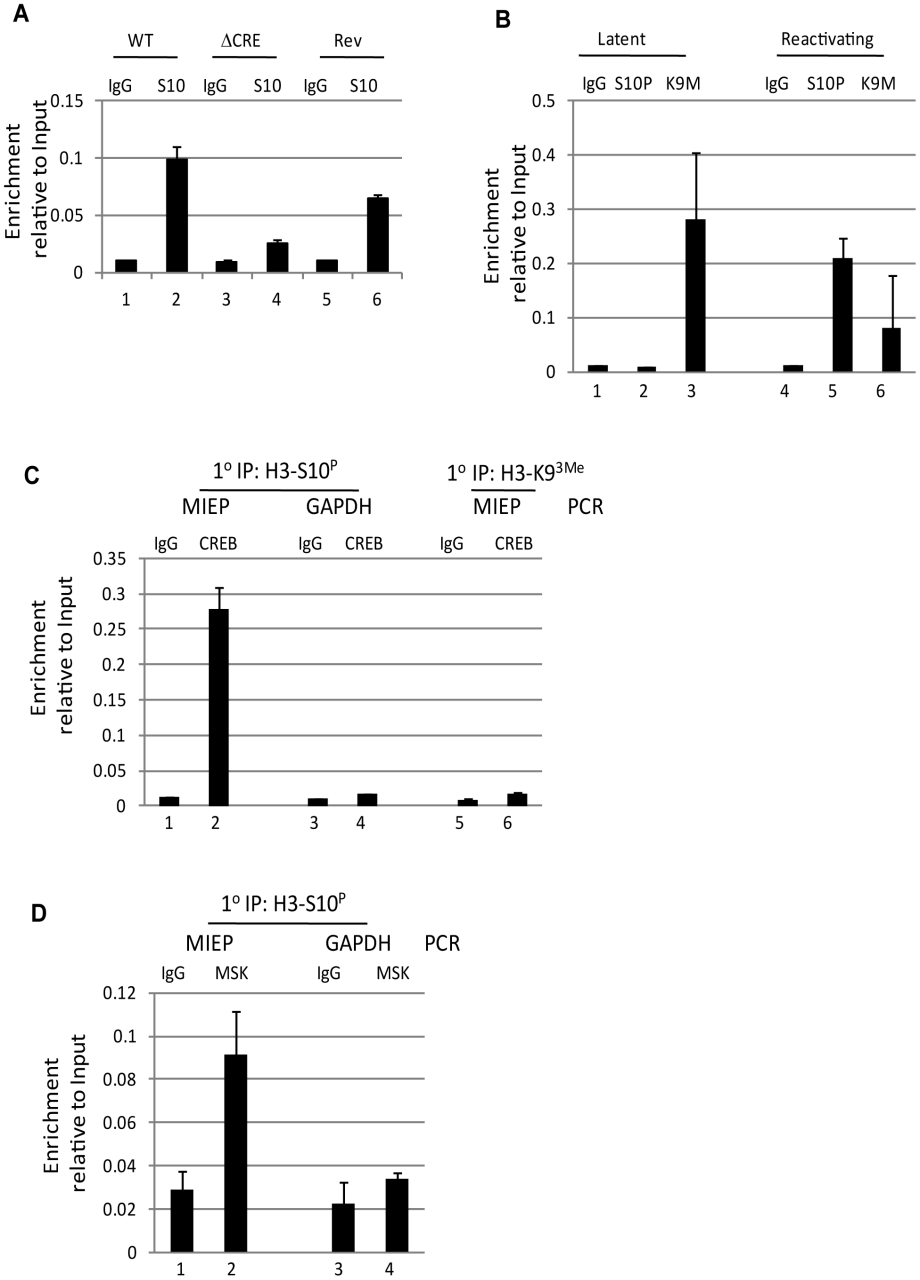
Phosphorylation of histone H3 is dependent on CREB binding to the MIEP. **A**) Immature DCs (iDC) derived from monocytes infected with wild type (WT), Revertant (Rev) or CRE deletion virus (ΔCRE) were left un-stimulated (1,3,5) or IL-6 treated cells (2,4,6). Then, chromatin immunoprecipitation of histone H3-S10^P^ was performed and samples amplified in an MIEP qPCR (2 hours post stimulation). Following normalisation to GAPDH, samples were expressed as a ratio of the Input signal. **B–C**) immature (latent;1,2) and IL-6 stimulated (reactivating, 3,4) DCs (2 hours post stimulation) were subject to chromatin immunoprecipitation with an anti-histone H3-S10^P^ (S10P), anti-histone H3-K9^3Me^ (K9M) or isoptype control (IgG) antibodies and then amplified in an MIEP qPCR and signal expressed as a ratio of the Input. **C**) The primary ChIPs from the reactivating samples (B) were then subject to a second ChIP with a phosphor CREB antibody or isotype matched control. The phosphor-CREB IP from H3-S10^P^ IP (1–4) was then expressed as a ratio of the Input signal for the MIEP or GAPDH qPCR. Alternatively, the phosphor-CREB or isotype control IPs from H3-K9^3Me^ (5,6) were amplified in an MIEP qPCR and expressed as a ratio of Input. S.D. of n = 2. **D**) IL-6 stimulated DCs (2 hours) were subject to a primary IP with anti-histone H3-serine 10 antibody then subject to second IP with an anti-MSK1 antibody or isoptype control. Samples were amplified in an MIEP or GAPDH promoter specific PCR and signal expressed as a ratio of Input. S.D. of n = 2.

Our data suggested that for the successful initiation of HCMV reactivation to occur phosphorylation of both CREB and histone H3 were necessary. Thus, we reasoned that ChIP analyses of reactivating cells generated from either wild type or ΔCRE infected monocytes could still be bound by phosphorylated histones and the defect in the ΔCRE virus possibly due to a lack of CREB binding. However, we were intrigued to observe that an overt lack of phosphorylated histone H3 bound to the MIEP of the ΔCRE infected cells was observed upon stimulation with IL-6 (Fig. 6A). This suggested that histone H3 phosphorylation at the MIEP was dependent on CREB binding. Consequently, we performed a ChIP re-ChIP analysis on reactivating cells to determine whether the binding of phosphorylated CREB was indeed co-incident with histone H3 phosphorylation. To do this we performed a ChIP analysis on IL-6 treated DCs 2 hours post stimulation or unstimulated DCs with anti-H3-S10^p^, anti-H3-K9^3Me^ and the appropriate isotype matched control. As expected, the analysis of the first round IP showed that the MIEPs in the stimulated DCs were predominantly associated with phosphorylated and not methylated histones whereas as in the unstimulated DCs the MIEP was predominantly associated with methylated histones (Fig. 6B) consistent with the IE gene expression profile in these different cell populations. We then focussed on the reactivating samples to determine whether CREB was concomitantly bound with H3-S10^P^. Consistent with our hypothesis, we could detect the IP of the MIEP following incubation with the p-CREB antibody. Crucially, a comparative analysis of the GAPDH promoter (the regulation of which is CREB-independent [47]) showed that this promoter was not enriched in the sample (Fig. 6C) nor did we detect any MIEP sequences in a p-CREB IP from the H3-K9^3Me^ primary IP sample. Taken together, these data suggest that phosphorylated CREB and histone H3-S10^P^ were concomitantly bound to the MIEP in IE expressing DC populations.

Finally, the data and the model predicted that MSK recruitment to the MIEP would also be on the same promoters harbouring phosphorylated histones. Thus we performed the same ChIP re-ChIP procedure to assess MSK binding. Our analysis, performed at 2 hpi (when both MSK and H3-S10-P can be detected on the MIEP (Figs. 5 and S5)) showed that we could observe enrichment for MSK binding to the MIEP also bound by H3-S10-P (Fig. 6D). Thus, MSK, CREB and H3-S10 phosphorylation could be detected on the same MIEPs in reactivating cells.

### A dominant negative form of ERK kinase, MEK1, inhibits IE gene expression

**Figure 7:**
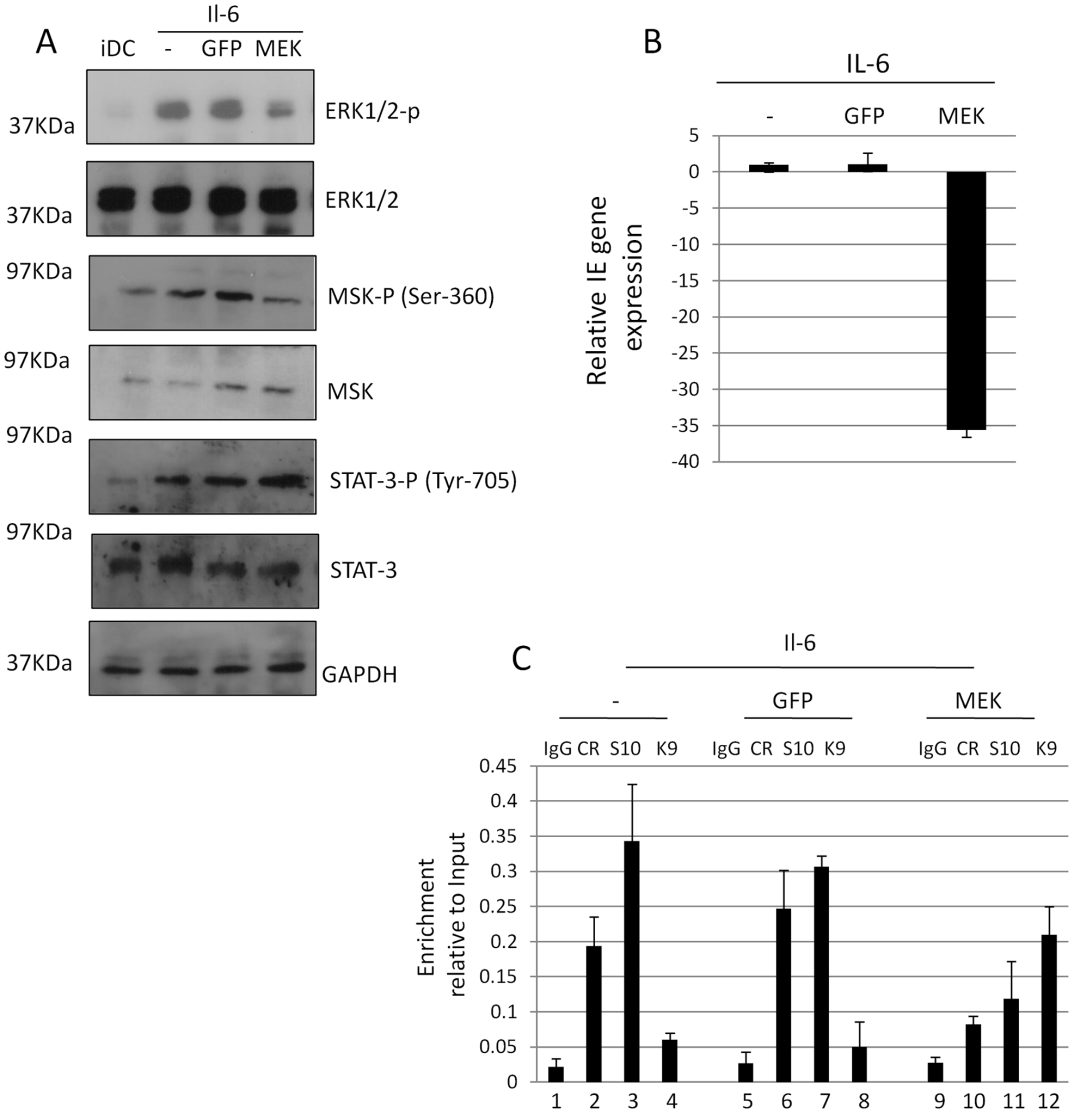
A dominant negative MEK1 blocks IE gene expression and CREB and histone H3 phosphorylation at the MIEP. **A**) Western blot analysis of iDCs (1), stimulated with IL-6 (2–4) post transduction with adenoviral vectors expressing GFP (3) or dn-MEK1 (4). **B**) RNA isolated from IL-6 stimulated iDCs pre infected with AD-GFP or AD-dnMEK was analysed by RT-qPCR for IE gene expression and expressed relative to IL-6 stimulated control. **C**) ChIP analyses were performed with isotype control (IgG), anti-phospho-CREB (CR), anti-phospho-serine 10 histone H3 (S10) or anti-trimethylated lysine 9 histone H3 (K9) antibodies and DNA amplified in an MIEP specific qPCR. Values were expressed relative to the Input control.

To address whether we could directly associate the known ERK-MSK relationship with CREB-histone H3 phosphorylation and, ultimately, the induction of IE gene expression from latency we utilised an adenovirus vector approach. We reasoned that the inefficient transfection of primary DCs with siRNAs would not allow us to see effects on HCMV when between 3–5% of cells are actively supporting HCMV latency based on IE positivity upon IL-6 stimulation. Therefore, we infected differentiating monocytes 4 days post differentiation with adenoviruses expressing GFP or a dominant negative form of ERK kinase MEK1 (Figure 7). Importantly, using adenoviruses we could establish between 60–70% infection based on the expression of GFP when the cells were analysed 24 hours post infection. Consistent with the expression of a dominant negative form MEK1, ERK and downstream phosphorylation of MSK was substantially reduced (Fig. 7A) – giving us further confidence that CREB phosphorylation in response to IL-6 was MSK and not PKA dependent (Fig. 5). Importantly, no effect on STAT-3 phosphorylation was observed in response to IL-6 confirming that there was not a global inhibition of IL-6 signalling (Fig. 7A). Subsequently, we analysed DCs 48 hours post infection with the adenoviruses for HCMV gene expression. Interestingly, expression of MEK1 had a profound impact on the level of IE gene expression in DCs (Fig. 7B). Pertinently, the expression of inactive MEK1 also impacted on CREB and histone H3 phosphorylation at the MIEP (Fig. 7C). The normal phosphorylation of CREB and histone H3, and concomitant loss of histone methylation, was severely abrogated (Fig. 7C).

### Activation of histone H3 phosphorylation induces IE gene expression in dendritic cells independent of CREB

**Figure 8:**
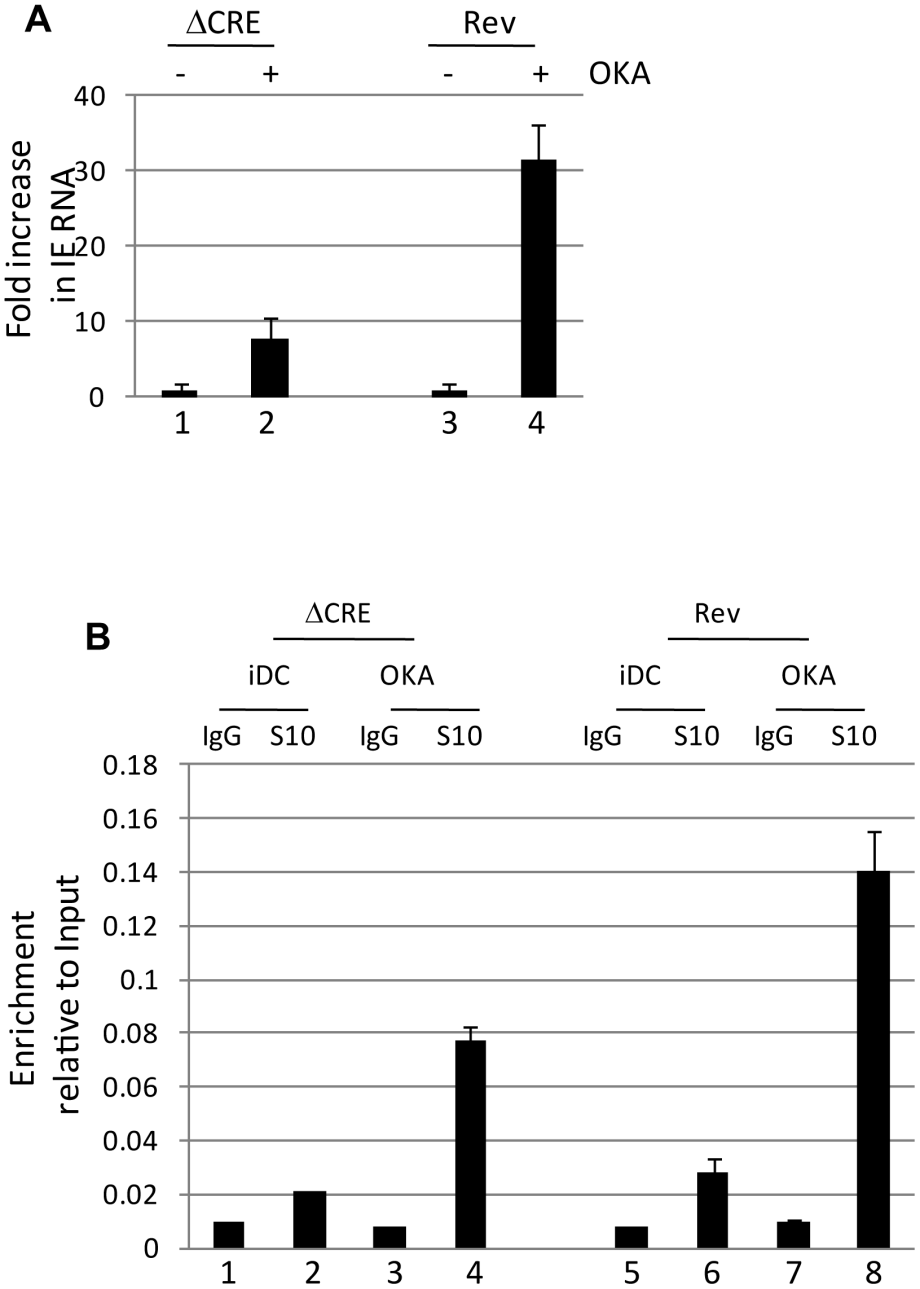
Okadaic acid promotes histone phosphorylation at the MIEP and partially rescuing the CRE deletion virus. **A**) Immature DCs (iDC) derived from monocytes infected with Revertant (Rev) or CRE deletion virus (ΔCRE) were treated with DMSO (1,3) or okadaic acid (2, 4) and amplified by qRT-PCR. IE expression was standardised to GAPDH (2^ΔCT^) and the fold change in gene expression (2^ΔΔCT^) following okadaic acid addition was expressed relative to the DMSO control. S.D of n = 3 **B**) Immature DCs (iDC) derived from monocytes infected with Revertant (Rev) or CRE deletion virus (ΔCRE) were treated with DMSO (iDC) or okadaic acid (OKA) and subject to ChIP with an anti-histone H3-S10^P^ antibody or isotype control. Samples were amplified in MIEP qPCRs and the signals expressed as a ratio of the Input. S.D. of n = 2.

The data thus far argue that MSK-mediated phosphorylation of CREB and histone H3 correlated with an induction of IE gene expression. We hypothesized that one explanation for the major role of CREB during reactivation is to enhance histone H3 phosphorylation which would promote gene expression via the adoption of an open chromatin conformation. We reasoned, therefore, that a surrogate signal for histone phosphorylation may be sufficient to trigger HCMV IE gene expression in the ΔCRE virus. Histone phosphorylation is dynamically regulated and, consequently, the inhibition of the histone dephosphorylase protein phosphatase 2A promotes hyper-phosphorylation of histones at a number of cellular promoters. Interestingly, incubation of immature DCs derived from latently infected monocytes with the protein phosphatase inhibitor okadaic acid was sufficient to drive induction of IE gene expression of both wild type and, albeit less efficiently, the ΔCRE virus (Fig. 8A). Although we cannot rule out off-target effects associated with the use of okadaic acid, the well established effects of okadaic acid on histone phosphorylation was consistent with elevated levels of phosphorylated histones bound to the MIEP were observed by ChIP assay correlating with the detection of HCMV gene expression (Fig. 8B) suggesting that chemical insults, similar to those proposed with the use of histone deacetylase inhibitors, triggered IE gene expression from quiescent virus. However, we note that unlike observed with HDAC inhibitors, okadaic acid did not trigger IE gene expression in monocytes under the same experimental conditions suggesting that cellular differentiation was also important.

## Discussion

Understanding the cellular and molecular basis of HCMV reactivation remains an enigmatic problem. Numerous studies of both HCMV and murine CMV have shown that inflammation is a key component of CMV reactivation [8], [13], [17], [18], [22], [24], [25] by inducing or augmenting the expression of the IE genes from a previously quiescent state. An understanding of the role of inflammation and, specifically, the signalling components activated by it, will be pivotal for delineation of the molecular basis of HCMV reactivation which, subsequently, impacts on the design of future therapeutic targets to control HCMV disease. Ultimately, a blockade – or at least a diminishment of the induction of IE gene expression will prevent efficient HCMV reactivation.

**Figure 9:**
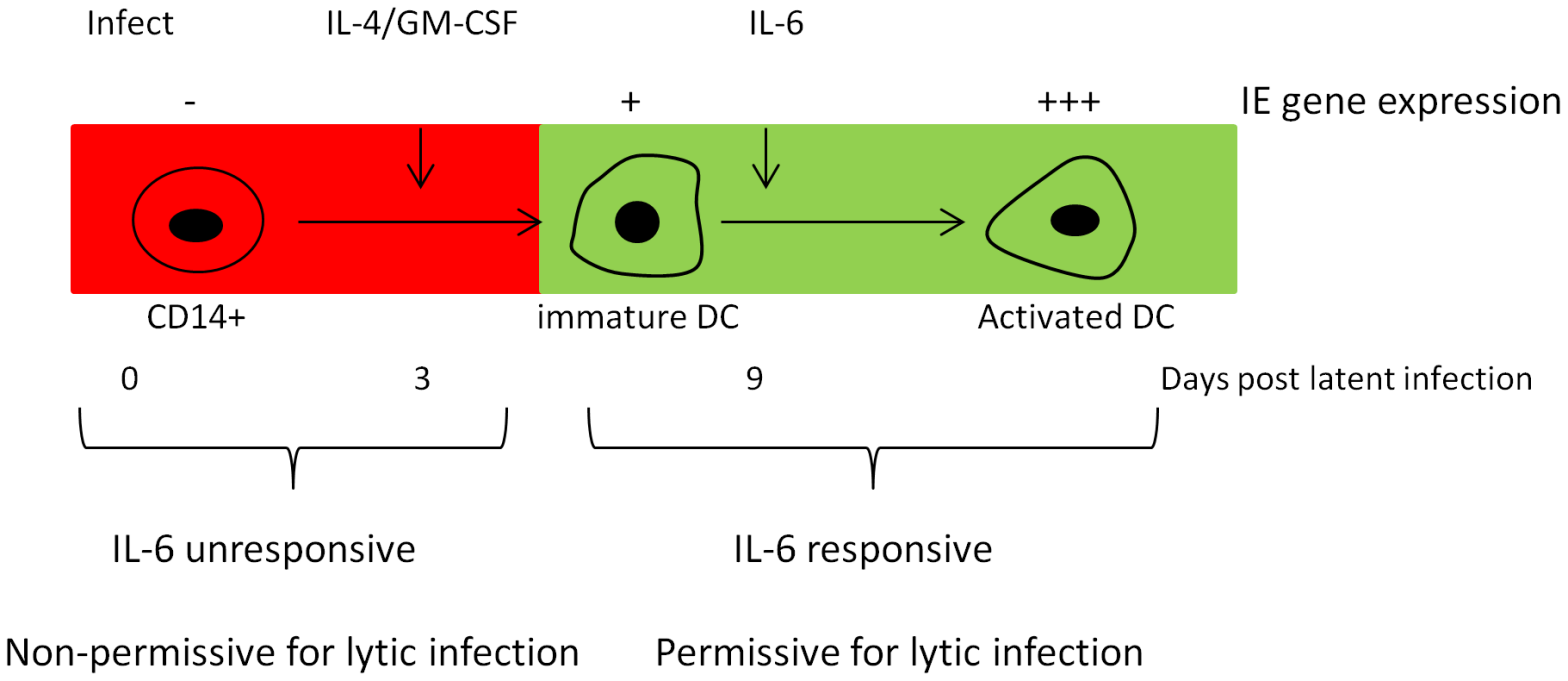
Model for the induction of IE gene expression in DCs. The expression of IE gene expression is restricted in CD14+ monocytes and is inherently unresponsive to inflammatory IL-6 signalling. However, cellular differentiation to a permissive phenotype renders latently infected cells responsive to stimuli that support IE gene expression and, ultimately, reactivation.

Pivotal early events in the reactivation of HCMV include cellular differentiation and concomitant activation of transcription of the major IE genes [1]. Indeed, we argue that cellular differentiation is a key event for the effects we observe. In our model for the induction of HCMV IE gene expression from latency we hypothesise that MSK activity via CREB is important for the first stages of HCMV reactivation in IL-6 stimulated immature DCs. Importantly, we also observe that IL-6 does not directly trigger HCMV reactivation from monocytes [13] and thus the MIEP in monocytes is intrinsically unresponsive to IL-6 (Fig. 9). This suggests that cellular differentiation, or even long term culture of monocytes [17], either eliminates an intrinsic block to IL-6 mediated activation of the MIEP or, alternatively, up-regulates a co-factor that helps drive the induction of HCMV gene expression from latency.

Robust IE gene expression can be triggered by IL-6 or LPS in this system and, previously, a number of different cytokines have been implicated in the initiation of HCMV and MCMV IE reactivation [8], [17], [18], [24], [25]. These include interferon gamma and TNF, interleukin-1β, or GM-CSF and IL-4 and thus cytokines important for myeloid cell differentiation and inflammation also promote and propagate CMV reactivation. Indeed, it is important to re-iterate that we argue that IL-6 induces robust reactivation of HCMV from immature DCs in our hands rather than acting as a ‘latency breaker’ – in that it cannot reverse latency in primitive progenitor cells. Consistent with this, low level reactivation is detectable in immature DCs generated using IL-4 and GM-CSF which we, and others, have reported previously [13], [17], [48] and could well be due to endogenous IL-6 production in the cultures by differentiating DCs or, even due to spontaneous maturation. However, the addition of exogenous IL-6 acts as a factor that drives robust HCMV IE gene expression in cells on the cusp of reactivation making it more efficient – with possible similarities to the ‘accelerator’ function recently postulated for the regulation of IE86 expression during lytic infection [49]. We reason that this could explain why neutralising IL-6 antibodies significantly reduce the level of HCMV reactivation we observe but do not abolish it completely.

It is highly plausible that there is not one molecular pathway that is responsible for acting as a latency breaker and that actually the notion of a single ‘latency breaker’ is fundamentally flawed. The composition of the MIEP should, theoretically, make it responsive to diverse stimuli. A number of motifs render it responsive to activators of NF-ĸB, CREB, AP-1 and IFN activated pathways suggesting that redundancy (or co-operativity) exists within the promoter. Consistent with redundancy, the deletion of a number of these elements does not significantly impact on the growth of HCMV mutants in fibroblasts in vitro [21]. In contrast, consistent with a degree of co-operativity, it has been reported that CREs located in the proximal enhancer region may be important for co-ordinating efficient transcription factor activity at the MIEP under specific circumstances [39]. As such, it has been postulated that these regions could be important in a cell type or context specific manner [20], [50]. In this study, we asked whether previously identified CRE elements were important for HCMV reactivation in primary cells. Based on these observations the reactivation of the ΔCRE virus was less efficient than wild type. This defect in reactivation correlated with a failure to promote the modification of histones from a latent (H3-K9^3me^) to an active (H3-K4^2me^) upon reactivation. Key to this switch was the activity of MSKs which promote extensive histone H3 phosphorylation at the MIEP in the early stages of reactivation. The idea of CREB/MSK-mediated phosphorylation of the histones bound to the MIEP as an important trigger of reactivation is highly attractive. Firstly, previous work has shown histone phosphorylation at serine 10 can synergise with histone acetylation [51] as well as de-stabilising the binding of chromo-domain proteins such as heterochromatin protein 1 [52], [53] – a protein that is recruited to methylated histones bound to promoters including the viral MIEP during latency [15]. Furthermore, histone phosphorylation is associated with a distinct set of promoters (the so-called ‘rapid response genes’) argued to promote hyper-acetylation of histones at these loci in a MAPK and MSK dependent manner [53]. Clearly, these events would positively influence HCMV reactivation if the MIEP is subject to the same mechanisms of regulation. Indeed, in vitro biochemical studies on the c-fos promoter have shown that the chromatin bound to the c-fos promoter is highly resistant to MSK mediated phosphorylation [54]. However, the binding of CREB (itself a target for MSK kinase activity) acts to promote histone phosphorylation and, as such, the parallels with our cell based analyses of HCMV latency and reactivation are striking. Furthermore, it is intriguing that this response at the c-fos promoter was enhanced by the presence of HMG (high mobility group) proteins [54] – a family of chromatin bound proteins implicated in the regulation of RTA mediated regulation of gamma herpes virus gene expression [55], [56] – including reactivation from latency. As such, it is tempting to speculate that MSK could play a role in the reactivation of multiple herpes viruses.

Initially it was slightly surprising that MSK but not RSK activity contributed to the reactivation. RSK activity is predominantly associated with ERK-MAPK signalling whereas MSKs are activated via both ERK and p38 mediated mechanisms yet it appears ERK activation is important for HCMV reactivation [13]. However, the role of ERK-MAPK signalling via MSKs in response to pro-inflammatory cytokine and growth factor stimulation [57] likely explains why this pathway predominates in the reactivation phenotype compared to other MAPK pathways in this setting. Furthermore, these data support the hypothesis that the MSK pathway provides important kinase activity responsible for histone phosphorylation in a CREB dependent manner at specific promoters. The detection of MSK binding to the MIEP prior to reactivation is supportive of this.

In conclusion, we have shown that IE gene expression from latency in DCs is acutely sensitive to MSK chemical inhibition and that reactivation of HCMV involves the phosphorylation of histones bound to the latent MIEP. MSK mediated phosphorylation of these histones is highly dependent on concomitant CREB binding to the MIEP. Indeed, it is an important consideration that these studies relied extensively on the use of chemical inhibitors which can have multiple effects on cellular signalling pathways over and above their reported specificities and thus will require further investigation. However, an interaction between CREB and MSK at the MIEP provides a mechanism for the signal integration required to promote HCMV reactivation from DCs in an ERK-MAPK dependent manner. Furthermore, using the regulation of the MIEP during HCMV latency and reactivation as a model of differential gene expression we provide further in vivo evidence for a functional interaction between MSK and CREB at promoters which, in this case, is important for controlling the gene expression of a clinically important pathogen. Clearly, because the effects of MSKs on gene expression are restricted to specific promoters further de-lineation of the pathways responsible for activation of the MIEP provides the potential for new therapeutic interventions.

## Materials and Methods

### Ethics statement

All research describing studies on primary human material with HCMV were assessed and approved by the Cambridge Local Research Ethics committee. Similarly, the collection of venous blood samples from anonymous donors was performed in accordance with established guidelines for the handling and processing of said tissue by the Cambridge Local Research Ethics committee.

### Virus, cell lines, culture and reagents

The clinical isolate TB40/e (a kind gift of Christian Sinzger, University of Tuebeingen, Germany) was purified from infected human retinal pigment epithelial cells using sorbitol gradients as previously described [58]. Viruses for these studies were characterised by their ability to infect primary dendritic cells to assay myelo-tropism – routinely, virus preparations infected 10–20% DCs when used at an MOI of 5 calculated on fibroblasts.

To generate HCMV mutants in the CREB response elements a galk BAC recombineering method was used as described previously [21]. Essentially, base substitutions in the 5 copies of CRE present in the MIEP was performed by site directed mutagenesis in the P4EΔM plasmid and the enhancer fragment excised using Eag1 and Bsrg1 restriction digest. For BAC mutagenesis, galk primers with 50 bp homology to HCMV MIE flanking +1 to −580 were used (5′- ATC TGA CGG TTC ACT AAA CGA GCT CTG CTT ATA TAG ACC TCC CAC CGT ACC CTG TTG ACA ATT AAT CAT CGG CA and 5′- CAG CGT GGA TGG CGT CTC CAG GCG ATC TGA CGG TTC ACT AAA CGC CTG TTG ACA ATT AAT CAT CGG CAT AGT ATA TCG GCA TAG) were used to amplify galk with flanking HCMV sequences using galk BAC in SW102. SW105 cells containing the TB40/e BAC were electroporated with the galk fragment and selected for using galactose containing agar plates and MacConkey galactose plates for verification. Sequence verified positive clones were then subject to a second round of recombination which introduced the Enhancer fragment isolated initially. Recombinants which had deleted galk were selected for on 2-deoxygalactose containing plates. Purified BACs were isolated, sequence verified and then transfected into permissive fibroblasts to reconstitute infectious virus.

The generation of the NF-ĸB mutant virus was performed using the same BAC mutagenesis protocol to generate point mutations by site-directed mutagenesis at positions −94, −157, −262 and −413 relative to +1 transcription start site in P4EΔM as described previously [59].

Primary CD14+ monocytes were isolated from the venous blood of anonymous donors who had given informed consent under the appropriate local rules. Typically, 50 mls of venous blood was diluted in PBS either 1∶1 and, following separation on a ficoll gradient (Lymphoprep, Nycomed, Melville, NY) peripheral blood mononuclear cells were then purified using either a CD14+ cell direct isolation kit (Miltenyi Biotec, Auburn, CA). Labelled CD14+ positive cells were rescued using Magnet Activated Cell Sorting (MACS) and cultured in X-vivo 15 (Cambrex, Walkersville, MD) supplemented with 2 mM L-Glutamine and cytokines to promote DC differentiation where appropriate.

### Experimental infection of primary cells and differentiation

The establishment of an in vitro experimental latent infection of primary CD14+ cells was performed by infecting CD14+ monocytes (5×10^5^ cells/well) were infected in 250 ul of X-vivo 15 media plus HCMV (MOI = 5) for 3 hours then rescued in 1 ml fresh media. After 3 days, experimentally infected CD14+ cells were differentiated to immature monocyte derived DCs (MoDCs) as described previously [13]. Briefly, CD14+ monocytes were cultured with Interleukin-4 (100 ng/ml) and GM-CSF (100 ng/ml) to promote differentiation to an interstitial-like DC phenotype [60]. Following culture, immature DCs was matured using Lipopolysaccharide (500 ng/ml; SIGMA, St. Louis, MO) or incubated with recombinant IL-6. All cytokines were from Peprotech (Rocky Hill, NJ) unless otherwise stated.

### Inhibitors and antibodies

To test for inhibition of HCMV reactivation, inhibitors were added prior to LPS or IL-6 stimulation. Inhibition of MEK/ERK was achieved using U0126 (or inactive analogue U0124; both 5 uM), tpl2 (tpl2 kinase Inhibitor II; 1 uM), Raf (Raf Kinase Inhibitor IV; 500 nM), RSK (BI-D1870; 500 nM; Selleckchem, Houston, TX) and MSK (H-89; 10 uM; Sigma Aldrich, Poole, UK) inhibitors (all from Calbiochem, San Diego, CA unless otherwise stated). All inhibitors were dissolved in DMSO and added 2 hours prior to addition of LPS directly to the culture media. Prior to use, all inhibitors were titrated to ensure determine a dose that gave activity against its target without any over effect on the viability of the cells (trypan blue exclusion). To promote histone H3 phosphorylation, the protein phosphatase 1 and 2A inhibitor okadaic acid (5 uM, Sigma Aldrich, Poole, UK) was added to cells 3 hours prior to the induction of reactivation. Where appropriate inhibitors were shown to have no impact on lytic infection of DCs or HFFs and, over the time frame of analyses, were shown not to be deleterious to cell viability.

For immuno-fluorescent detection of viral antigens, cells were fixed in 4% paraformaldehyde and then permeabilised with 0.1% Triton X/PBS. An anti-IE (Millipore, 1∶1000 in PBS) and then with an Alexafluor 594 nm conjugated Goat anti-mouse antibody (Millipore; 1∶1000 in PBS). All antibody incubations were performed for 1 hour at room temperature.

Protein expression was detected by Western blotting of denatured protein samples. To detect protein expression nitrocellulose filters were incubated at 4°C overnight with anti-CREB or anti-phosphor-CREB antibodies, (1∶1000; Upstate Biotechnology, Charlottesville, VA), anti-p42/p44 or phosphor-anti-p42/p44 antibodies, anti-MSK1 or anti-phosphor-MSK1 (serine 360) (all 1∶1000; Cell Signalling Technology, Danvers, MA) and anti-STAT-3 or phosphor-STAT-3 (tyrosine 705) (1∶250; Santa Cruz Biotechnology, Santa Cruz, CA) and then incubated with a HRP conjugated Goat anti-rabbit secondary antibody (1∶4000; Santa Cruz Biotechnology, Santa Cruz, CA) for 1 hr at RT. As a loading control, nitrocellulose filters were also incubated with a rabbit anti-GAPDH control (1∶1000; Abcam, Cambridge, UK) followed by an HRP conjugated goat anti-rabbit antibody (1∶4000; Santa Cruz Biotechnology, Santa Cruz, CA) – both antibody incubations performed at RT for 1 hr. Blots were processed using ECL plus (GE life sciences, Amersham, UK) and exposed to Kodak autoradiograph film (Sigma-Aldrich, Poole, UK).

### Nucleic acid isolation, reverse transcription and PCR

RNA was isolated from 10^6^ cells using RNAeasy spin columns as described by the manufacturer (Qiagen, Valencia, CA). Following isolation, total RNA was incubated with DNAse I (Promega, Madison, WI) and then amplified by qRT-PCR. IE, UL138 and GAPDH gene expression was determined using a Taqman Triplex qRT-PCR system (SIGMA, Poole, UK) with the following primers and probes: IE, 5′- CAA GAA CTC AGC CTT CCC TAA GAC and 5′- TGA GGC AAG TTC TCG AAT GC with the probe CCA ATG GCT GCA GTC AGG CCA TG-(TAM); UL138, 5′- CGC TGT TTC TCT GGT TAG and 5′- CAG ACG ATA CCG TTT CTC with the probe CCG ACG ACG AAG ACG ATG AAC-(Cy5); and GAPDH, 5′- GGA AGC TTG TCA TCA ATG and 5′- CCC CAC TTG ATT TTG GAG with the probe ATC ACC ATC TTC CAG GAG CGA G-(JOE). Reactions were set up using the Qiagen RT-PCR Quantitect kit (Qiagen, Sussex, UK) in accordance with the manufacturer's protocol and the samples amplified for ABI 7500 Fast Real Time PCR machine (Applied Biosystems, Foster City, CA; 95°C for 15 s and 60°C for 45 s).

### Chromatin immunoprecipitation and analysis

All procedures were performed essentially as described previously. Briefly, DCs were fixed with 1% formaldehyde (10 mins) and then lysed and sonicated to fragment DNA. DNA associated with histones was immunoprecipitated with control serum (Sigma, Poole, UK), anti-phosphor-histone H3-serine 10 antiserum (ChIP grade, 1∶200 dilution), anti-dimethyl lysine 4 histone H3 antiserum (ChIP grade, 1∶200), anti-trimethyl lysine 9 H3 antiserum (ChIP grade, 1∶200 dilution), anti-phosphor or total CREB (both 1∶250), or anti-MSK1 antibody (1∶200 dilution; Cell signalling Technology, Danvers, MA) - all antibodies Upstate Biotechnology, Charlottesville, VA unless stated. For detection of the MIEP of HCMV, DNA from disrupted nucleosomes was precipitated and amplified by qPCR using 5′-CCAAGTCTCCACCCCATTGAC (sense) and 5′-GACATTTTGGAAAGTCCCGTTG (anti-sense) primers and quantified using a Taqman probe FAM-5′-TGGGAGTTTGTTTTGGCACCAAA-3′-TMR. Cellular DNA sequences (GAPDH promoter) were amplified using 5′-CGGCTACTAGCGGTTTTACG (sense) and 5′-AAGAAGATGCGGCTGACTGT (anti-sense) and quantified using a Taqman probe FAM-5′-CACGTAGCTCAGGCCTCAAGACCT-3′-TMR. Specific immuno-precipitation of sequences was expressed as enrichment from Input.

### Adenoviral delivery of MEK1 proteins and analysis in latency

To deliver exogenous proteins to primary cells, adenoviral vectors expressing GFP or a dominant negative version of MEK1, were used (Cell Biolabs, San Diego, CA). Four days into the 6 day differentiation protocol, differentiating cells were infected with adenoviral expression vectors (MOI 250) for 3 hours then the media replaced with fresh growth media. At Day 6 of differentiation (48 hours post adenovirus infection), cells were stimulated with IL-6 and then harvested for Western Blot analysis 15 mins post culture, 2 hours post stimulation for ChIP analyses, or left overnight and subject to either RT-qPCR for IE gene expression.

## Supporting Information

Figure S1 **CREB does not bind to the MIEP of the ΔCRE virus.** Chromatin immunoprecipitations on immature DCs (iDC) stimulated with IL-6 (2 hours) were performed with an anti-CREB antibody or isotype control. Samples were amplified in an MIEP qPCR and expressed as a ratio of the Input. Error bars represent SD of a triplicate analysis.
(PDF)

Figure S2 **Deletion of CRE from the MIEP does not impact on infection of DCs.** monocyte derived DCs or HFFs were infected with wild type, ΔCRE or revertant viruses (MOI = 5:HFFs) and stained, 24 hpi, for IE expression and counter-stained with DAPI. Cell counts from 5 random fields were performed from triplicate wells.
(PDF)

Figure S3 **Deletion of the NF-kB binding sites does not inhibit IL-6 induced reactivation in DCs.** **A**) Monocytes infected with wild type (WT), Revertant (Rev) or ΔNF-kB deletion virus (ΔNF-kB) were analysed 5 days post infection for UL138 and GAPDH gene expression by qRT-PCR. Viral gene expression (UL138) was expressed as a ratio to GADPH. **B**) Alternatively, infected CD14 cells were differentiated to immature DCs and stimulated with IL-6 to promote reactivation. The percentage of IE positive cells was calculated by indirect immunofluorescence and DAPI nuclear counterstaining. S.D. shown from n = 2 (A,B).
(PDF)

Figure S4 **The inhibition of HCMV reactivation by IL-6 neutralisation occur immediately post LPS stimulation.** **A**) CD14+ cells infected with HCMV were differentiated to immature DCs and then stimulated with LPS (2–6) to induce reactivation. Cells were then incubated with neutralising IL6 antibodies (3–6) between 0–6 hours post LPS stimulation and percentage reactivation calculated by indirect immunofluorescence for IE gene expression (24 hrs) and DAPI nuclear counterstaining. S.D. shown from n = 3.
(PDF)

Figure S5 **Histone H3 phosphorylation at serine 10 accumulates post IL-6 stimulation of DCs.** Chromatin immunopreciptations with anti-histone H3 phospho-serine 10 or isotype control antibodies were performed on DCs derived from monocytes infected with HCMV (1–3) subsequently stimulated with IL-6 between 15 minutes and 3 hours post reactivation. DNA was amplified in an MIEP PCR and expressed as ratio of the Input sample. S.D. of n = 2.
(PDF)

Figure S6 **Inhibition of Raf-ERK MAPK signalling blocks phosphor- and acetyl modification of histones at the MIEP.** **A–B**) Chromatin immunoprecipitation of histone H3-S10^P^, pan acetyl histone H4 or isotype matched control was performed on CD14+ cells differentiated to immature DCs. Prior to IL-6 (A) or LPS (B) stimulation cells were incubated with DMSO, ERK, Raf or tpl2 inhibitors for 2 hours then stimulated for 3 hours prior to ChIP. DNA was then amplified in an MIEP qPCR and signal expressed as a ratio of the Input. S.D. of n = 2.
(PDF)

Figure S7 **Histone phosphorylation occurs independent of viral gene expression.** **A–B**) Immature DCs derived from monocytes infected HCMV were incubated with actinomycin D (4 hours pre-stimulation) and then incubated with IL-6. At 2 hours post stimulation ChIP assays (A) with anti-histone H3-phospho serine 10 or isotype control antibodies was performed. DNA was amplified in an MIEP qPCR and signals expressed as a ratio of Input S.D. n = 2. Alternatively, RNA was isolated 24 hours post IL-6 stimulation and analysed for IE and GAPDH gene expression by qPCR (B). The fold increase in IE gene expression was calculated relative to immature DCs not stimulated with IL-6. S.D. n = 2.
(PDF)
