# Correction: Mitogen and Stress Activated Kinases Act Co-operatively with CREB during the Induction of Human Cytomegalovirus Immediate-Early Gene Expression from Latency

**DOI:** 10.1371/journal.ppat.1004392

**Published:** 2014-09-05

**Authors:** The PLOS Pathogens Staff

Panel A in Figure 5 contains an error. Some of the western blot lanes taken from Figure 2C were incorrectly flipped and inserted into Figure 5A. The Figure legend is correct and shown here for reference. Please see the corrected version of Figure 5 here.

**Figure 5:**
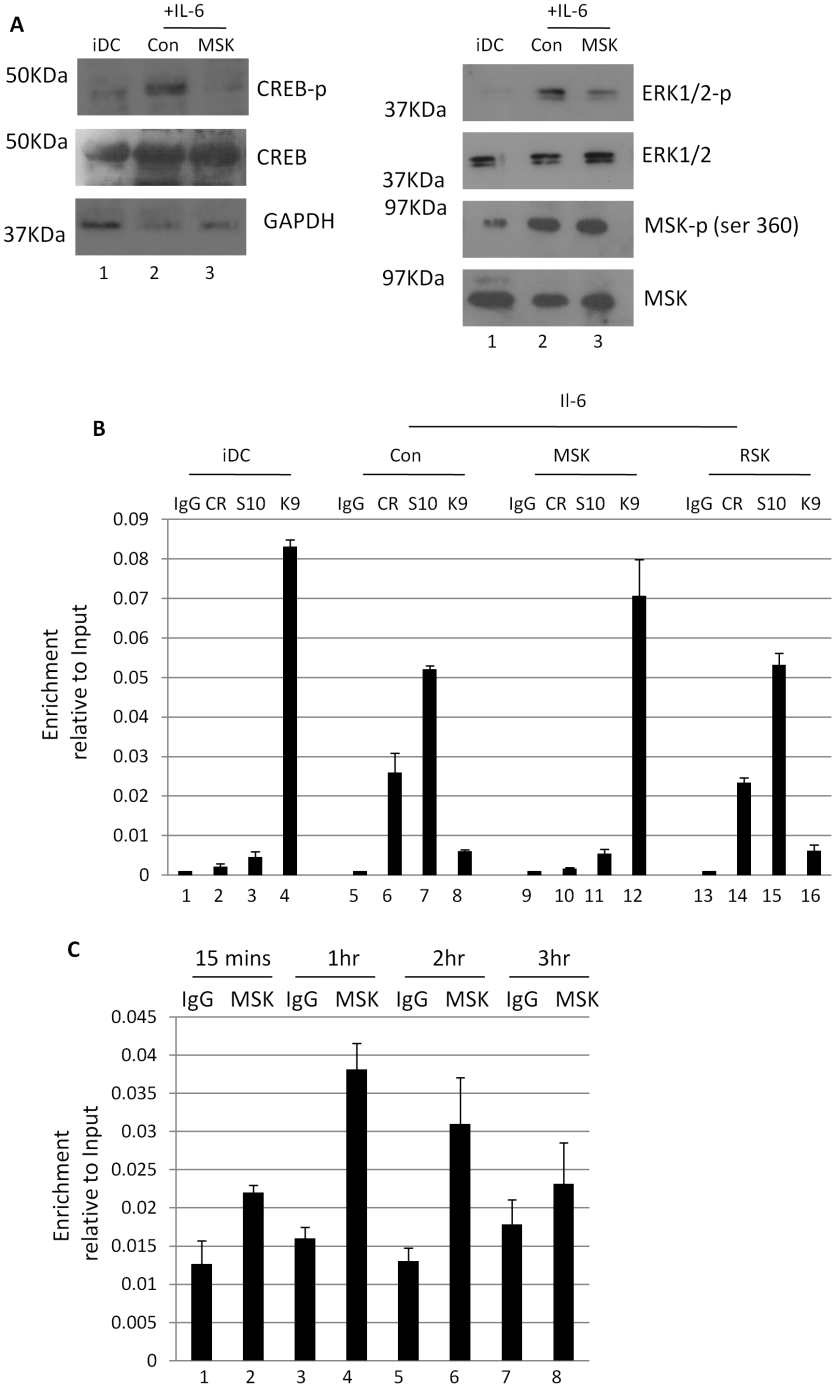
Inhibition of mitogen and stress kinase activity blocks CREB and histone phosphorylation at the MIEP. **A**) Western blot for phosphor and total CREB, phosphor and total ERK1/2, phosphor and total MSK and GAPDH was performed on immature DCs or DCs stimulated with IL-6 (30 mins) after incubation with DMSO or MSK inhibitor (2 hours). **B**) Chromatin immunopreciptations on immature DCs (iDC) derived from monocytes infected with HCMV (1–3) were performed alongside IL-6 (4–12) stimulated DCs for phosphor-CREB (CR), histone H3-S10^P^ (S10) and histone H3-K9^3Me^ (K9) binding at 2 hours post stimulation. DNA was amplified in an MIEP PCR and expressed as ratio of the Input sample. S.D. of n  =  2. **C**) Chromatin immunopreciptations on immature DCs (iDC) stimulated with IL-6 were performed at 15 mins to 3 hours post stimulation with an anti-MSK antibody or an isotype matched control. DNA was amplified in an MIEP PCR and expressed as ratio of the Input sample. S.D. of n  =  2.

## Supporting Information

The supporting information files originally provided in the manuscript were corrupted. We have replaced Figures S1-S7 with the correct figure files. The original PDF file was also corrupted, but has now been fixed. All of these files are now downloadable from the original article
