# Supplementary figures and images for: Mitogen and Stress Activated Kinases Act Co-operatively with CREB during the Induction of Human Cytomegalovirus Immediate-Early Gene Expression from Latency
Source: PLoS Pathog. 2014 Jun 12;10(6):e1004195. doi: 10.1371/journal.ppat.1004195 (PMC4055774; doi:10.1371/journal.ppat.1004195)

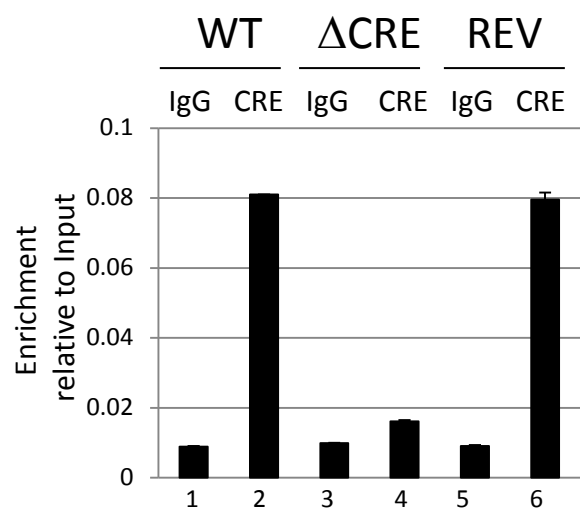

Figure S1

Supplement: Figure S1 — CREB does not bind to the MIEP of the ΔCRE virus. Chromatin immunoprecipitations on immature DCs (iDC) stimulated with IL-6 (2 hours) were performed with an anti-CREB antibody or isotype control. Samples were amplified in an MIEP qPCR and expressed as a ratio of the Input. Error bars represent SD of a triplicate analysis. (PDF) [file ppat.1004195.s001.pdf]

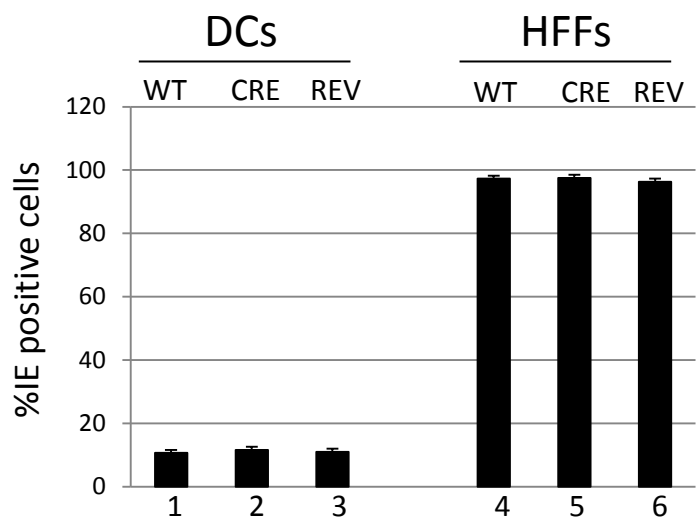

Figure S2

Supplement: Figure S2 — Deletion of CRE from the MIEP does not impact on infection of DCs. monocyte derived DCs or HFFs were infected with wild type, ΔCRE or revertant viruses (MOI = 5:HFFs) and stained, 24 hpi, for IE expression and counter-stained with DAPI. Cell counts from 5 random fields were performed from triplicate wells. (PDF) [file ppat.1004195.s002.pdf]

**A**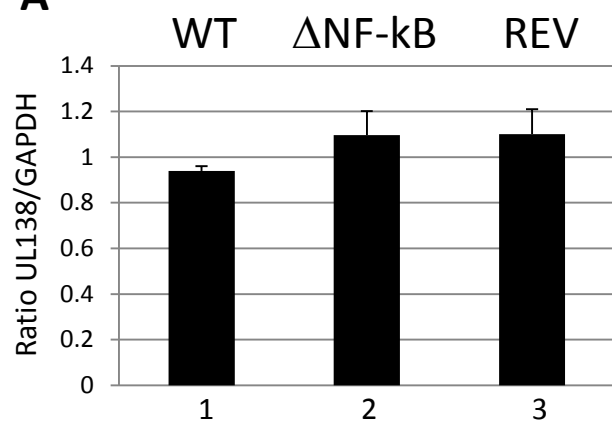**B**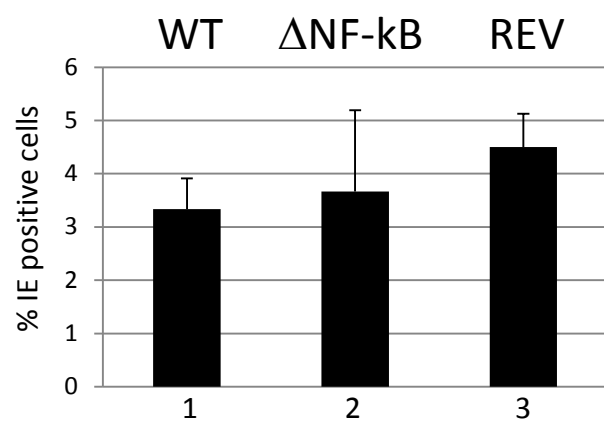

Supplement: Figure S3 — Deletion of the NF-kB binding sites does not inhibit IL-6 induced reactivation in DCs. A) Monocytes infected with wild type (WT), Revertant (Rev) or ΔNF-kB deletion virus (ΔNF-kB) were analysed 5 days post infection for UL138 and GAPDH gene expression by qRT-PCR. Viral gene expression (UL138) was expressed as a ratio to GADPH. B) Alternatively, infected CD14 cells were differentiated to immature DCs and stimulated with IL-6 to promote reactivation. The percentage of IE positive cells was calculated by indirect immunofluorescence and DAPI nuclear counterstaining. S.D. shown from n = 2 (A,B). (PDF) [file ppat.1004195.s003.pdf]

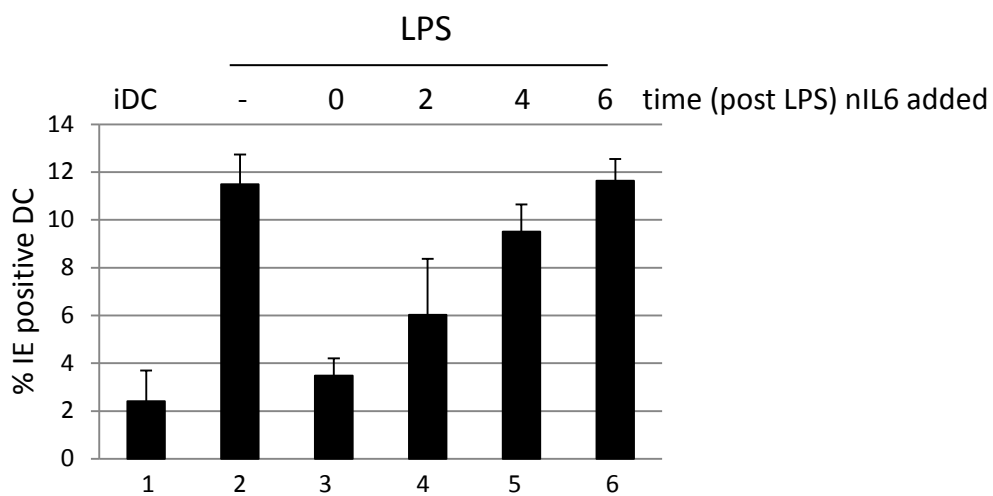

Figure S4

Supplement: Figure S4 — The inhibition of HCMV reactivation by IL-6 neutralisation occur immediately post LPS stimulation. A) CD14+ cells infected with HCMV were differentiated to immature DCs and then stimulated with LPS (2–6) to induce reactivation. Cells were then incubated with neutralising IL6 antibodies (3–6) between 0–6 hours post LPS stimulation and percentage reactivation calculated by indirect immunofluorescence for IE gene expression (24 hrs) and DAPI nuclear counterstaining. S.D. shown from n = 3. (PDF) [file ppat.1004195.s004.pdf]

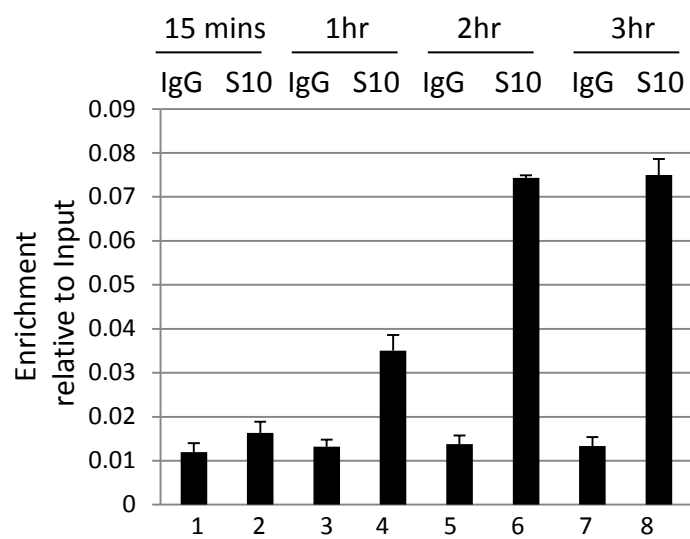

Figure S5

Supplement: Figure S5 — Histone H3 phosphorylation at serine 10 accumulates post IL-6 stimulation of DCs. Chromatin immunopreciptations with anti-histone H3 phospho-serine 10 or isotype control antibodies were performed on DCs derived from monocytes infected with HCMV (1–3) subsequently stimulated with IL-6 between 15 minutes and 3 hours post reactivation. DNA was amplified in an MIEP PCR and expressed as ratio of the Input sample. S.D. of n = 2. (PDF) [file ppat.1004195.s005.pdf]

LPS

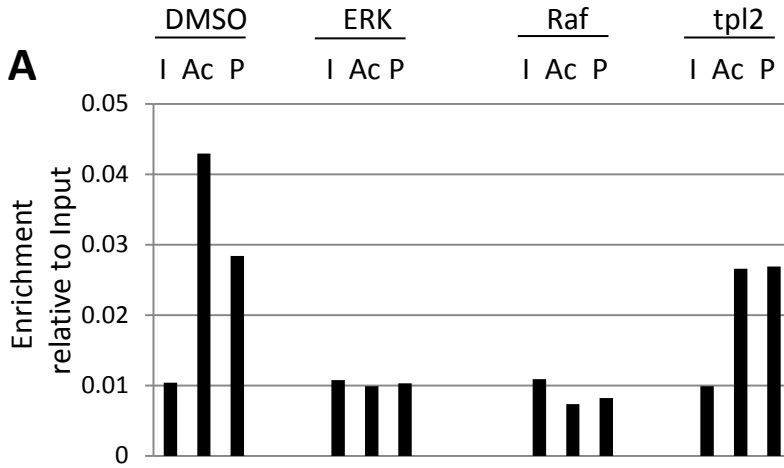

IL-6

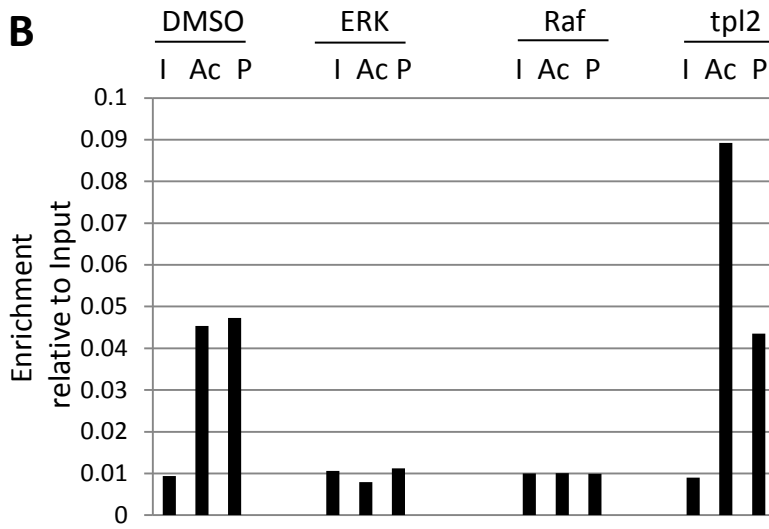

Supplement: Figure S6 — Inhibition of Raf-ERK MAPK signalling blocks phosphor- and acetyl modification of histones at the MIEP. A–B) Chromatin immunoprecipitation of histone H3-S10P, pan acetyl histone H4 or isotype matched control was performed on CD14+ cells differentiated to immature DCs. Prior to IL-6 (A) or LPS (B) stimulation cells were incubated with DMSO, ERK, Raf or tpl2 inhibitors for 2 hours then stimulated for 3 hours prior to ChIP. DNA was then amplified in an MIEP qPCR and signal expressed as a ratio of the Input. S.D. of n = 2. (PDF) [file ppat.1004195.s006.pdf]

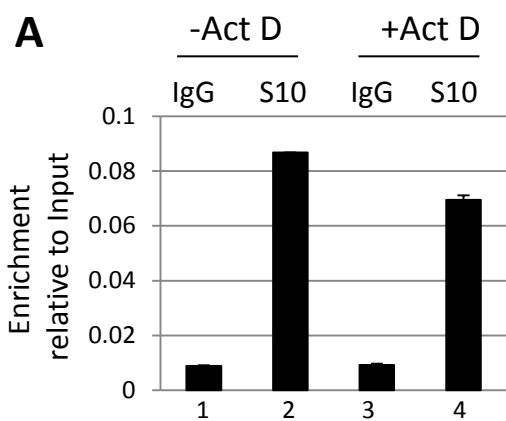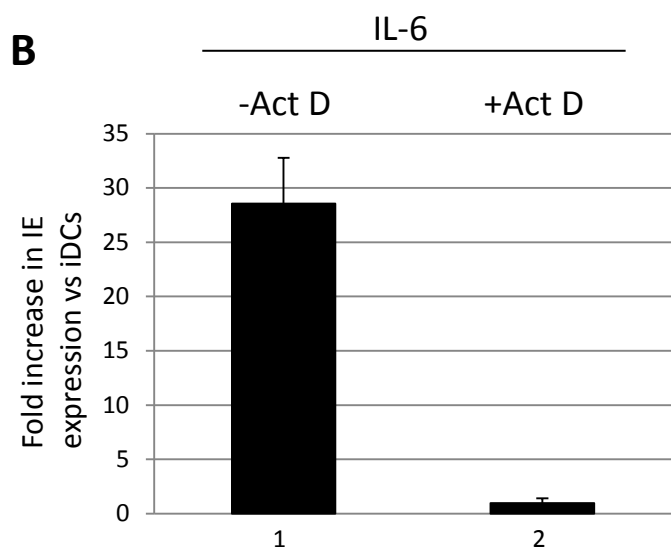

Figure S7

Supplement: Figure S7 — Histone phosphorylation occurs independent of viral gene expression. A–B) Immature DCs derived from monocytes infected HCMV were incubated with actinomycin D (4 hours pre-stimulation) and then incubated with IL-6. At 2 hours post stimulation ChIP assays (A) with anti-histone H3-phospho serine 10 or isotype control antibodies was performed. DNA was amplified in an MIEP qPCR and signals expressed as a ratio of Input S.D. n = 2. Alternatively, RNA was isolated 24 hours post IL-6 stimulation and analysed for IE and GAPDH gene expression by qPCR (B). The fold increase in IE gene expression was calculated relative to immature DCs not stimulated with IL-6. S.D. n = 2. (PDF) [file ppat.1004195.s007.pdf]
